# The Strategic Role of Nursing in Implementing CureAll in the Americas: Essential Competencies and Workforce Strengthening^1^

**DOI:** 10.1590/1518-8345.0000.4991

**Published:** 2026-04-27

**Authors:** Luís Carlos Lopes-Júnior, Monnie Abraham, Edmara Bazoni Soares Maia, Maurício Maza, María Liliana Vásquez Ponce, Regina Aparecida Garcia de Lima

**Affiliations:** 1 Pan American Health Organization, Department of Noncommunicable Diseases and Mental Health, Washington, DC, United States of America.; 2 Universidade Federal do Espírito Santo, Departamento de Enfermagem, Vitória, ES, Brazil.; 3 St Jude Children’s Research Hospital, Department of Global Pediatric Medicine, Memphis, Tennessee, United States of America.; 4 Universidade Federal de São Paulo, Escola Paulista de Enfermagem, São Paulo, SP, Brazil.; 5 University of São Paulo at Ribeirão Preto College of Nursing, Departament of Maternal Infant Nursing and Public Health, Ribeirão Preto, SP, Brazil.

**Figure:**
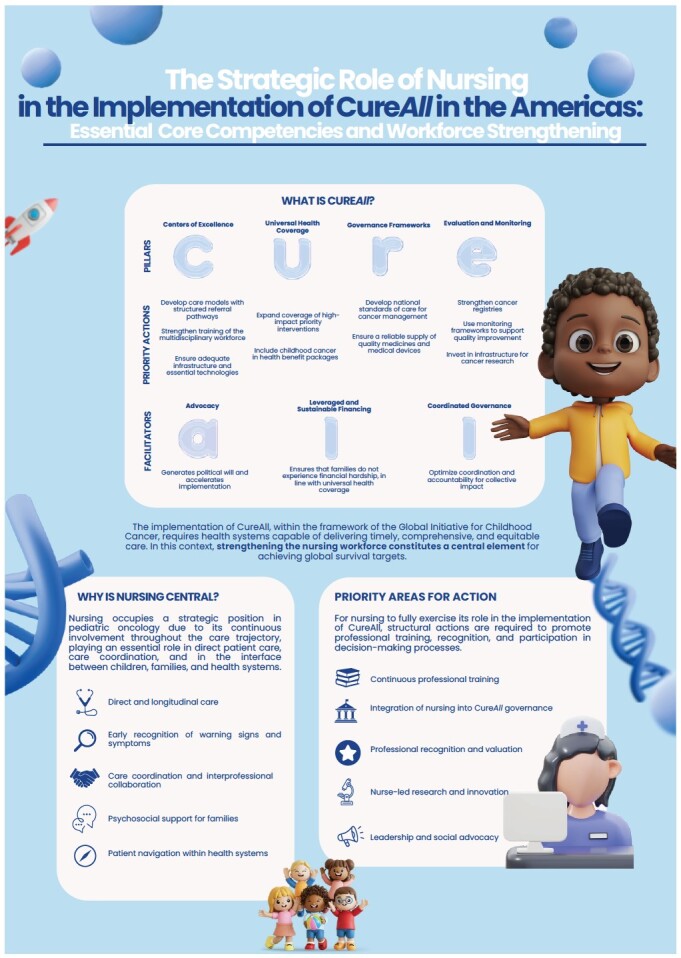

**Figure:**
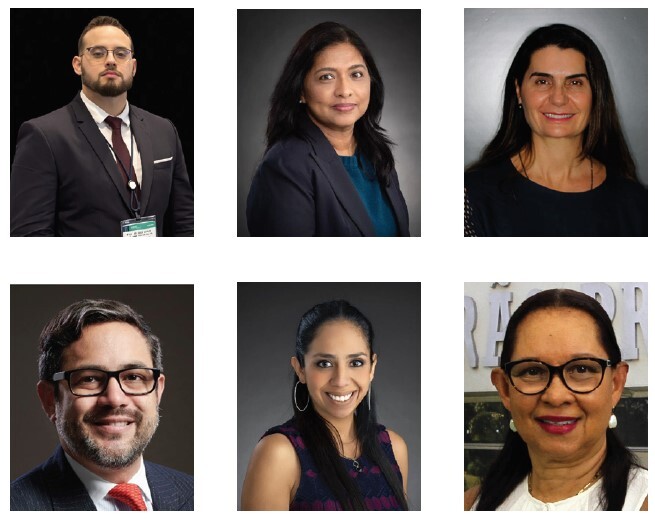

The Global Initiative for Childhood Cancer (GICC), launched by the World Health Organization (WHO) in 2018, aims to increase global survival rates for childhood cancer to at least 60% by 2030 by strengthening health systems and integrating pediatric oncology care into national cancer control agendas^1^. To operationalize this goal, the CureAll Framework was developed, structuring actions into four pillars and three essential enablers, proposing a strategic model to improve access, quality of care, and equity for children and adolescents with cancer^1^ ^-^ ^2^. The four pillars include: (i) C - Centers of excellence and integrated care networks; (ii) U - Universal health coverage; (iii) R - Regimens and standards for clinical management, including access to essential medicines and health technologies; and (iv) E - Evaluation, monitoring, and health information systems. These pillars are supported by three cross-cutting enablers: A - advocacy; L - leveraged and sustainable financing; and L - aligned governance and interinstitutional coordination, considered fundamental for the successful and equitable implementation of the initiative across different national contexts^1^.

Among the main challenges identified in low- and middle-income countries are delayed diagnosis, fragmented access to care, the lack of standardized protocols, and the shortage of qualified professionals^3^ ^-^ ^4^. The nursing workforce plays a central role across all these areas, contributing to direct care as well as care coordination, triage, health education, and psychosocial support. However, the strategic integration of Pediatric Oncology Nursing into national and regional action plans remains limited, particularly in Latin America and the Caribbean (LAC)^4^.

In recent years, the Pan American Health Organization (PAHO) and the World Health Organization, in partnership with institutions such as St. Jude Children’s Research Hospital, have led a regional effort to strengthen pediatric oncology care, promoting technical products aligned with the CureAll Framework. One example is the publication produced by the RN-Latin America Pediatric Oncology Nursing Expert Panel - an international consortium of pediatric oncology nurses, pediatric nurses, oncology-certified nurses, and oncologists engaged with the GICC - entitled Pediatric Oncology Nursing Practice in Latin America and the Caribbean^5^. This publication systematizes the scope of practice and proposes essential competencies for Pediatric Oncology Nursing in the region, grounded in a robust scoping review and aligned with internationally recognized frameworks^4^ ^-^ ^5^.

Nursing should be recognized as a strategic agent in the implementation of CureAll due to its capillarity, close relationship with families, and central role in longitudinal care. The development of clinical, communication, cultural, and managerial competencies - particularly those related to the early recognition of warning signs, pediatric palliative care, toxicity management, and treatment adherence - is crucial to achieving the objectives proposed by WHO^3^ ^-^ ^5^.

This strategic role is also aligned with the WHO document Global Strategic Directions for Nursing and Midwifery 2021-2025^6^, which defines four priority areas of action to enable nurses and midwives to fully contribute to universal health coverage and the Sustainable Development Goals (SDGs): education, jobs, leadership, and service delivery. Investing in competency-based education, creating and retaining jobs, expanding nurses’ leadership in decision-making bodies, and ensuring safe and decent practice environments are essential conditions for the effective implementation of CureAll in the Americas^6^.

Furthermore, the Essential Competencies for Pediatric Oncology Nursing in Latin America and the Caribbean, recently systematized into six domains (clinical and supportive care; education and research; engagement and advocacy; interprofessional collaboration; leadership and professional development; and health policy and service development), provide a structured framework to guide clinical practice, education, and health system strengthening in the region^5^. These domains emerged from a comprehensive synthesis of evidence and reflect both the diversity of approaches and the recurring core themes that underpin pediatric oncology nursing practice^5^.

The delineation of these domains highlights the dynamic nature of oncology nursing practice, which integrates direct care for children, adolescents, and their families with broader responsibilities in education, research, and policy development. In this sense, the scope of practice should be understood as the set of functions, responsibilities, and activities that professionals are qualified to perform safely, ethically, and with quality, based on competencies acquired through formal education, continuing professional development, and clinical experience. Recognizing Pediatric Oncology Nursing as a subspecialty requiring specific and ongoing preparation promotes not only professional recognition but also the capacity to impact clinical outcomes, quality of life, and equity in care throughout the region^3^ ^-^ ^5^.

In this context, the RN-Latin America Pediatric Oncology Nursing Expert Panel^6^ proposes five priority areas for action, focusing on the strategic role of nursing in implementing CureAll in the Americas:

- Continuous professional training: expand continuing education programs with content on early detection, treatment toxicities, pediatric palliative care, and family-centered approaches.
- Integration of nursing into CureAll governance: ensure the active participation of nurses in national committees and coordination networks for pediatric oncology as experts and policy contributors.
- Professional recognition and appreciation: promote salary policies, labor protection, emotional well-being, and institutional recognition of the critical role of pediatric oncology nurses.
- Nurse-led research and innovation: encourage studies on treatment adherence, social barriers, symptom science, precision nursing, self-care interventions, and the use of technologies to improve clinical outcomes.
- Leadership and social advocacy: position nurses as advocates for the rights of children and adolescents with cancer and their families, influencing policymakers and society to strengthen oncology care policies.

In Brazil, pediatric oncology care is guaranteed comprehensively and free of charge by the Unified Health System (SUS). Recently, in 2024, the Ministry of Health relaunched CureAll Brazil, in alignment with the WHO Global Initiative for Childhood Cancer. The event included government officials and health authorities, signaling political and technical commitment to implementing the global goals of the CureAll Framework^7^. This movement is directly aligned with the Sustainable Development Goals, particularly SDG 3 (Good Health and Well-being) by seeking to reduce childhood cancer mortality; SDG 10 (Reduced Inequalities) by addressing ethnic and regional disparities in access to diagnosis and treatment; and SDG 9 (Industry, Innovation, and Infrastructure) by fostering the development of scientific and technological solutions such as genomic precision medicine panels applicable to the Brazilian context^1^ ^-^ ^4^.

Thus, it is imperative to: a) institutionalize essential Pediatric Oncology Nursing competencies in undergraduate and graduate curricula; b) systematically include nurses in governance committees and multidisciplinary teams responsible for implementing CureAll; c) expand nurse-led scientific production in low- and middle-income settings; d) recognize and strengthen the role of nursing in national childhood cancer control plans; and e) ensure continuing education for nurses working in primary, secondary, and tertiary care, focusing on early detection, integrated care, and humanization of health care.

Equity in pediatric and adolescent oncology care will only be possible with the strengthening of a well-trained, well-distributed, and valued nursing workforce. The transformation proposed by CureAll will only be achieved through structural investments in nursing and its recognition as a central actor in addressing childhood cancer in the Americas. The future of personalized, effective, and humanized care necessarily lies in the hands of nursing.
