# Supplementary material for: The Strategic Role of Nursing in Implementing CureAll in the Americas: Essential Competencies and Workforce Strengthening
Source: Rev Lat Am Enfermagem. 2026 Apr 27;34:e4991. doi: 10.1590/1518-8345.0000.4991 (PMC13124038; doi:10.1590/1518-8345.0000.4991)
Supplement: Translation

# Translation: O papel estratégico da Enfermagem na implementação do *CureAll* nas Américas: competências essenciais e fortalecimento da força de trabalho^2^

**DOI:** 10.1590/1518-8345.0000.4992

**Authors:** Luís Carlos Lopes-Júnior, Monnie Abraham, Edmara Bazoni Soares Maia, Maurício Maza, María Liliana Vásquez Ponce, Regina Aparecida Garcia de Lima

**Affiliations:** 1 Pan American Health Organization, Department of Noncommunicable Diseases and Mental Health, Washington, DC, United States of America.; 2 Universidade Federal do Espírito Santo, Departamento de Enfermagem, Vitória, ES, Brazil.; 3 St Jude Children’s Research Hospital, Department of Global Pediatric Medicine, Memphis, Tennessee, United States of America.; 4 Universidade Federal de São Paulo, Escola Paulista de Enfermagem, São Paulo, SP, Brazil.; 5 University of São Paulo at Ribeirão Preto College of Nursing, Departament of Maternal Infant Nursing and Public Health, Ribeirão Preto, SP, Brazil.

A Iniciativa Global para o Câncer Infantil (GICC), lançada pela Organização Mundial da Saúde (OMS) em 2018, tem como meta elevar as taxas globais de sobrevida para o câncer infantil a, pelo menos, 60% até 2030, por meio do fortalecimento dos sistemas de saúde e da integração do cuidado oncológico pediátrico nas agendas nacionais de controle do câncer^1^. Para operacionalizar essa meta, foi desenvolvido o *CureAll Framework*, que estrutura ações em quatro pilares e três facilitadores essenciais, propondo um modelo estratégico para melhorar o acesso, a qualidade do cuidado e a equidade para crianças e adolescentes com câncer^1^ ^-^ ^2^. Os quatro pilares compreendem: (i) C - Centros de excelência e redes integradas de atenção; (ii) U - Cobertura universal em saúde; (iii) R - Regimes e padrões de manejo clínico, incluindo o acesso a medicamentos essenciais e tecnologias em saúde; e (iv) E - Sistemas de avaliação, monitoramento e informação em saúde. Esses pilares são sustentados por três facilitadores transversais: A - *advocacy*; L - financiamento alavancado e sustentável; e L - governança articulada e coordenação interinstitucional, considerados fundamentais para a implementação bem-sucedida e equitativa da iniciativa em diferentes contextos nacionais^1^.

Entre os principais desafios identificados nos países de baixa e média renda, destacam-se o diagnóstico tardio, a fragmentação no acesso, a carência de protocolos padronizados e a escassez de profissionais qualificados^3^ ^-^ ^4^. A força de trabalho da Enfermagem desempenha papel central em todos esses eixos, atuando tanto na atenção direta quanto na coordenação do cuidado, na triagem, na educação em saúde e no suporte psicossocial. No entanto, ainda é limitada a inserção estratégica da Enfermagem Oncológica Pediátrica nos planos nacionais e regionais de ação, especialmente na América Latina e Caribe (ALC)^4^.

Nos últimos anos, a Organização Pan-Americana de Saúde e a Organização Mundial de Saúde, em parceria com instituições como o *St. Jude Children’s Research Hospital*, têm liderado um esforço regional para fortalecer o cuidado oncológico pediátrico, promovendo produtos técnicos alinhados ao *CureAll Framework*. Um exemplo é a publicação, produzida pelo *RN-Latin America Pediatric Oncology Nursing Expert Pane*l - um consórcio internacional de enfermeiros de oncologia pediátrica, enfermeiros pediátricos, enfermeiros certificados em oncologia e oncologistas engajados com a GIIC - denominado *Pediatric Oncology Nursing Practice in Latin America and the Caribbean* ^5^, que sistematiza o escopo de prática e propõe competências essenciais para a Enfermagem Oncológica Pediátrica na região, fundamentada em uma robusta revisão de escopo e alinhada a referenciais internacionais reconhecidos^4^ ^-^ ^5^.

A Enfermagem deve ser vista como agente estratégica na implementação do *CureAll* por sua capilaridade, proximidade com as famílias e papel central no cuidado longitudinal. O desenvolvimento de competências clínicas, comunicacionais, culturais e gerenciais - especialmente voltadas para o reconhecimento precoce de sinais de alerta, cuidados paliativos, manejo da toxicidade e adesão ao tratamento - é crucial para alcançar os objetivos propostos pela OMS^3^ ^-^ ^5^. 

Esse papel estratégico se alinha também ao documento da OMS *Global Strategic directions for Nursing and Midwifery* 2021-2025^6^, que define quatro áreas prioritárias de ação para que enfermeiros e parteiras contribuam plenamente para a cobertura universal de saúde e os Objetivos de Desenvolvimento Sustentável (ODS): educação, empregos, liderança e prestação de serviços. Investir em educação baseada em competências, criar e reter postos de trabalho, ampliar a liderança de enfermeiros em instâncias decisórias e garantir ambientes de prática seguros e dignos constitui condição indispensável para que o *CureAll* seja efetivamente implementado nas Américas^6^.

Além disso, as Competências Essenciais da Enfermagem Oncológica Pediátrica na América Latina e Caribe, recentemente sistematizadas em seis domínios (atenção clínica e de suporte; educação e pesquisa; engajamento e *advocacy;* trabalho interprofissional; liderança e desenvolvimento profissional; e desenvolvimento de políticas e serviços de saúde), oferecem um marco de referência estruturado para orientar a prática clínica, a formação e o fortalecimento dos sistemas de saúde na região^5^. Esses domínios resultaram de uma síntese abrangente de evidências e refletem tanto a diversidade de abordagens quanto os temas centrais recorrentes que sustentam a atuação da Enfermagem em Oncologia Pediátrica^5^.

O delineamento desses domínios destaca a natureza dinâmica da prática da Enfermagem Oncológica, que integra o cuidado direto às crianças, aos adolescentes e suas famílias com responsabilidades mais amplas em educação, pesquisa e formulação de políticas. Nesse sentido, o escopo da prática deve ser compreendido como o conjunto de funções, responsabilidades e atividades que os profissionais estão aptos a desempenhar com segurança, ética e qualidade, a partir de competências adquiridas por meio de formação, educação permanente e experiência clínica. Ao reconhecer a Enfermagem Oncológica Pediátrica como uma subespecialidade que exige preparo específico e contínuo, promove-se não apenas a valorização profissional, mas também a capacidade de impactar desfechos clínicos, qualidade de vida e equidade no cuidado em toda a região^3^ ^-^ ^5^.

Nesse contexto, o *RN-Latin America Pediatric Oncology Nursing Expert Panel* ^6^ propõe cinco eixos prioritários de ação, com foco no papel estratégico da Enfermagem na implementação do *CureAll* nas Américas:

- Capacitação profissional contínua: ampliar programas de educação permanente, com conteúdos sobre detecção precoce, toxicidades, cuidados paliativos pediátricos e abordagens centradas na família.
- **Inserção da Enfermagem na governança do *CureAll*:**garantir a participação ativa de enfermeiros em comitês nacionais e redes de coordenação da oncologia pediátrica, como especialistas e formuladores de políticas.
- Reconhecimento e valorização profissional: promover políticas salariais, proteção laboral, equilíbrio emocional e reconhecimento institucional à atuação crítica dos enfermeiros oncológicos pediátrico.
- Pesquisa e inovação lideradas por enfermeiros: incentivar estudos sobre adesão ao tratamento, barreiras sociais, ciência dos sintomas, enfermagem de precisão, intervenções de autocuidado e uso de tecnologias para melhorar desfechos clínicos.
- **Liderança e *advocacy* social:**posicionar enfermeiros como defensores dos direitos das crianças e dos adolescentes com câncer e de suas famílias, influenciando gestores e a sociedade para o fortalecimento das políticas de cuidado oncológico.

No Brasil, o cuidado oncológico pediátrico é garantido de forma integral e gratuita pelo Sistema Único de Saúde, e recentemente, em 2024, o Ministério da Saúde relançou o *CureAll* Brasil, em sintonia com a Iniciativa Global para o Câncer Infantil da OMS. O evento contou com a presença de gestores governamentais e autoridades sanitárias sinalizando um compromisso político e técnico com a implementação das metas globais do *CureAll Framework* ^7^. Esse movimento se articula diretamente com os Objetivos de Desenvolvimento Sustentável, em especial com o ODS 3 (Saúde e Bem-estar), ao buscar reduzir a mortalidade infantojuvenil por câncer; com o ODS 10 (Redução das Desigualdades), ao enfrentar disparidades étnicas e regionais no acesso ao diagnóstico e tratamento e com o ODS 9 (Inovação e infraestrutura), ao fomentar o desenvolvimento de soluções científicas e tecnológicas, como painéis genômicos de medicina de precisão aplicáveis ao contexto brasileiro^1^ ^-^ ^4^.

Assim, é imperativo: a) a** **institucionalização das competências essenciais da Enfermagem Oncológica Pediátrica nos currículos da graduação e pós-graduação; b) a** **inclusão sistemática de enfermeiros nos comitês gestores e equipes multidisciplinares** **responsáveis pela implementação do*CureAll*; c) a** **ampliação da produção científica liderada por enfermeiros** **em contextos de baixa e média renda; d) o reconhecimento e fortalecimento do papel da Enfermagem nos planos nacionais de controle do câncer infantojuvenil e e) a** **garantia de educação permanente para enfermeiros da atenção primária, secundária e terciária**,** com foco em detecção precoce, cuidados integrados e humanização do cuidado em saúde.

A equidade no cuidado oncológico pediátrico e juvenil só será possível com o fortalecimento de uma força de trabalho de Enfermagem bem formada, bem distribuída e valorizada. A transformação proposta pelo *CureAll* só será possível com investimentos estruturais na Enfermagem e seu reconhecimento como ator central no enfrentamento do câncer infantojuvenil nas Américas. O futuro do cuidado personalizado, efetivo e humanizado passa, necessariamente, pelas mãos da Enfermagem.

# Translation: **El papel estratégico de la Enfermería en la implementación del *CureAll* en las Américas: competencias esenciales y fortalecimiento de la fuerza laboral** ^3^

**DOI:** 10.1590/1518-8345.0000.4990

**Authors:** Luís Carlos Lopes-Júnior, Monnie Abraham, Edmara Bazoni Soares Maia, Maurício Maza, María Liliana Vásquez Ponce, Regina Aparecida Garcia de Lima

**Affiliations:** 1 Pan American Health Organization, Department of Noncommunicable Diseases and Mental Health, Washington, DC, United States of America.; 2 Universidade Federal do Espírito Santo, Departamento de Enfermagem, Vitória, ES, Brazil.; 3 St Jude Children’s Research Hospital, Department of Global Pediatric Medicine, Memphis, Tennessee, United States of America.; 4 Universidade Federal de São Paulo, Escola Paulista de Enfermagem, São Paulo, SP, Brazil.; 5 University of São Paulo at Ribeirão Preto College of Nursing, Departament of Maternal Infant Nursing and Public Health, Ribeirão Preto, SP, Brazil.

La Iniciativa Global para el Cáncer Infantil (GICC), lanzada por la Organización Mundial de la Salud (OMS) en 2018, tiene como objetivo aumentar las tasas globales de supervivencia del cáncer infantil al menos al 60% para el año 2030, mediante el fortalecimiento de los sistemas de salud y la integración del cuidado oncológico pediátrico en las agendas nacionales de control del cáncer^1^. Para operacionalizar este objetivo, se desarrolló 

el *CureAll Framework*, que estructura acciones en cuatro pilares y tres facilitadores esenciales, proponiendo un modelo estratégico para mejorar el acceso, la calidad de la atención y la equidad para niños, niñas y adolescentes con cáncer^1^ ^-^ ^2^. Los cuatro pilares comprenden: (i) C - Centros de excelencia y redes integradas de atención; (ii) U - Cobertura universal de salud; (iii) R - Regímenes y estándares de manejo clínico, incluyendo el acceso a medicamentos esenciales y tecnologías sanitarias; y (iv) E - Sistemas de evaluación, monitoreo e información en salud. Estos pilares se sustentan en tres facilitadores transversales: A - abogacía (*advocacy*); L - financiamiento apalancado y sostenible; y L - gobernanza articulada y coordinación interinstitucional, considerados fundamentales para la implementación exitosa y equitativa de la iniciativa en diferentes contextos nacionales^1^.

Entre los principales desafíos identificados en los países de ingresos bajos y medianos se destacan el diagnóstico tardío, la fragmentación en el acceso, la falta de protocolos estandarizados y la escasez de profesionales calificados^3^ ^-^ ^4^. La fuerza laboral de Enfermería desempeña un papel central en todos estos ejes, actuando tanto en la atención directa como en la coordinación del cuidado, el triaje, la educación en salud y el apoyo psicosocial. Sin embargo, la inserción estratégica de la Enfermería Oncológica Pediátrica en los planes nacionales y regionales de acción sigue siendo limitada, especialmente en América Latina y el Caribe (ALC)^4^.

En los últimos años, la Organización Panamericana de la Salud (OPS) y la Organización Mundial de la Salud, en asociación con instituciones como el *St. Jude Children’s Research Hospital*, han liderado un esfuerzo regional para fortalecer la atención oncológica pediátrica, promoviendo productos técnicos alineados con el *CureAll Framework*. Un ejemplo es la publicación producida por el *RN-Latin America Pediatric Oncology Nursing Expert Panel* - un consorcio internacional de enfermeros de oncología pediátrica, enfermeros pediátricos, enfermeros certificados en oncología y oncólogos comprometidos con la GICC - titulada *Pediatric Oncology Nursing Practice in Latin America and the Caribbean* ^5^. Esta publicación sistematiza el alcance de la práctica y propone competencias esenciales para la Enfermería Oncológica Pediátrica en la región, fundamentada en una sólida revisión de alcance y alineada con marcos internacionales reconocidos^4^ ^-^ ^5^.

La Enfermería debe ser reconocida como un agente estratégico en la implementación del *CureAll* debido a su capilaridad, su cercanía con las familias y su papel central en el cuidado longitudinal. El desarrollo de competencias clínicas, comunicacionales, culturales y gerenciales - especialmente orientadas al reconocimiento temprano de signos de alerta, los cuidados paliativos pediátricos, el manejo de toxicidades y la adherencia al tratamiento - es crucial para alcanzar los objetivos propuestos por la OMS^3^ ^-^ ^5^.

Este papel estratégico también se alinea con el documento de la OMS *Global Strategic Directions for Nursing and Midwifery* 2021-2025^6^, que define cuatro áreas prioritarias de acción para que enfermeros y parteras contribuyan plenamente a la cobertura universal de salud y a los Objetivos de Desarrollo Sostenible (ODS): educación, empleo, liderazgo y prestación de servicios. Invertir en educación basada en competencias, crear y retener puestos de trabajo, ampliar el liderazgo de enfermería en instancias decisorias y garantizar entornos de práctica seguros y dignos constituye una condición indispensable para la implementación efectiva del *CureAll* en las Américas^6^.

Además, las Competencias Esenciales de la Enfermería Oncológica Pediátrica en América Latina y el Caribe, recientemente sistematizadas en seis dominios (atención clínica y de apoyo; educación e investigación; compromiso y abogacía; trabajo interprofesional; liderazgo y desarrollo profesional; y desarrollo de políticas y servicios de salud), ofrecen un marco de referencia estructurado para orientar la práctica clínica, la formación y el fortalecimiento de los sistemas de salud en la región^5^. Estos dominios surgieron de una síntesis integral de la evidencia y reflejan tanto la diversidad de enfoques como los temas centrales recurrentes que sustentan la práctica de la enfermería en oncología pediátrica^5^.

La delimitación de estos dominios destaca la naturaleza dinámica de la práctica de la Enfermería Oncológica, que integra el cuidado directo a los niños, los adolescentes y sus familias con responsabilidades más amplias en educación, investigación y formulación de políticas. En este sentido, el alcance de la práctica debe comprenderse como el conjunto de funciones, responsabilidades y actividades que los profesionales están capacitados para desempeñar con seguridad, ética y calidad, a partir de competencias adquiridas mediante la formación, la educación permanente y la experiencia clínica. Al reconocer la Enfermería Oncológica Pediátrica como una subespecialidad que exige una preparación específica y continua, se promueve no solo la valorización profesional, sino también la capacidad de impactar los desenlaces clínicos, la calidad de vida y la equidad en la atención en toda la región^3^ ^-^ ^5^.

En este contexto, el *RN-Latin America Pediatric Oncology Nursing Expert Panel* ^6^ propone cinco ejes prioritarios de acción, con énfasis en el papel estratégico de la Enfermería en la implementación del *CureAll* en las Américas:

- Capacitación profesional continua: ampliar programas de educación permanente, con contenidos sobre detección precoz, toxicidades, cuidados paliativos pediátricos y enfoques centrados en la familia.
- **Inserción de la Enfermería en la gobernanza del *CureAll*:** garantizar la participación activa de enfermeros en comités nacionales y redes de coordinación de la oncología pediátrica, como especialistas y formuladores de políticas.
- Reconocimiento y valorización profesional: promover políticas salariales, protección laboral, equilibrio emocional y reconocimiento institucional al papel crítico de los enfermeros oncológicos pediátricos.
- Investigación e innovación lideradas por enfermeros: incentivar estudios sobre adherencia al tratamiento, barreras sociales, ciencia de los síntomas, enfermería de precisión, intervenciones de autocuidado y uso de tecnologías para mejorar los desenlaces clínicos.
- **Liderazgo y *advocacy* social:** posicionar a los enfermeros como defensores de los derechos de los niños y adolescentes con cáncer y de sus familias, influyendo en gestores y en la sociedad para fortalecer las políticas de atención oncológica.

En Brasil, la atención oncológica pediátrica está garantizada de forma integral y gratuita por el Sistema Único de Salud, y recientemente, en 2024, el Ministerio de Salud relanzó el CureAll Brasil, en consonancia con la Iniciativa Global para el Cáncer Infantil de la OMS. El evento contó con la presencia de gestores gubernamentales y autoridades sanitarias, lo que señaló un compromiso político y técnico con la implementación de las metas globales del CureAll Framework^7^. Este movimiento se articula directamente con los Objetivos de Desarrollo Sostenible, especialmente con el ODS 3 (Salud y Bienestar), al buscar reducir la mortalidad infantil y juvenil por cáncer; con el ODS 10 (Reducción de las Desigualdades), al enfrentar las disparidades étnicas y regionales en el acceso al diagnóstico y tratamiento; y con el ODS 9 (Innovación e infraestructura), al fomentar el desarrollo de soluciones científicas y tecnológicas, como paneles genómicos de medicina de precisión aplicables al contexto brasileño^1^ ^-^ ^4^.

Así, es imperativo: a) la institucionalización de las competencias esenciales de la Enfermería Oncológica Pediátrica en los currículos de grado y posgrado; b) la inclusión sistemática de enfermeros en los comités de gestión y equipos multidisciplinarios responsables de la implementación del *CureAll*; c) la ampliación de la producción científica liderada por enfermeros en contextos de países de ingresos bajos y medianos; d) el reconocimiento y fortalecimiento del papel de la Enfermería en los planes nacionales de control del cáncer infantil y juvenil; y e) la garantía de educación permanente para enfermeros de la atención primaria, secundaria y terciaria, con énfasis en la detección precoz, los cuidados integrados y la humanización de la atención en salud.

La equidad en el cuidado oncológico pediátrico y adolescente solo será posible con el fortalecimiento de una fuerza laboral de Enfermería bien formada, bien distribuida y valorada. La transformación propuesta por el *CureAll* solo será posible con inversiones estructurales en la Enfermería y su reconocimiento como actor central en el enfrentamiento del cáncer infantil y adolescente en las Américas. El futuro del cuidado personalizado, efectivo y humanizado pasa, necesariamente, por las manos de la Enfermería.
